# Supplementary material for: Splice-Junction-Based Mapping of Alternative Isoforms in the Human Proteome
Source: Cell Rep. Author manuscript; Available in PMC 2020 Jan 15. (PMC6961840; doi:10.1016/j.celrep.2019.11.026)
Supplement: 2 [file NIHMS1546469-supplement-2.zip › DF1/PXD006675/CardiacFibroblast/CardiacFibroblast_25_RFWD2_QLEAQLEQIQK.pdf]

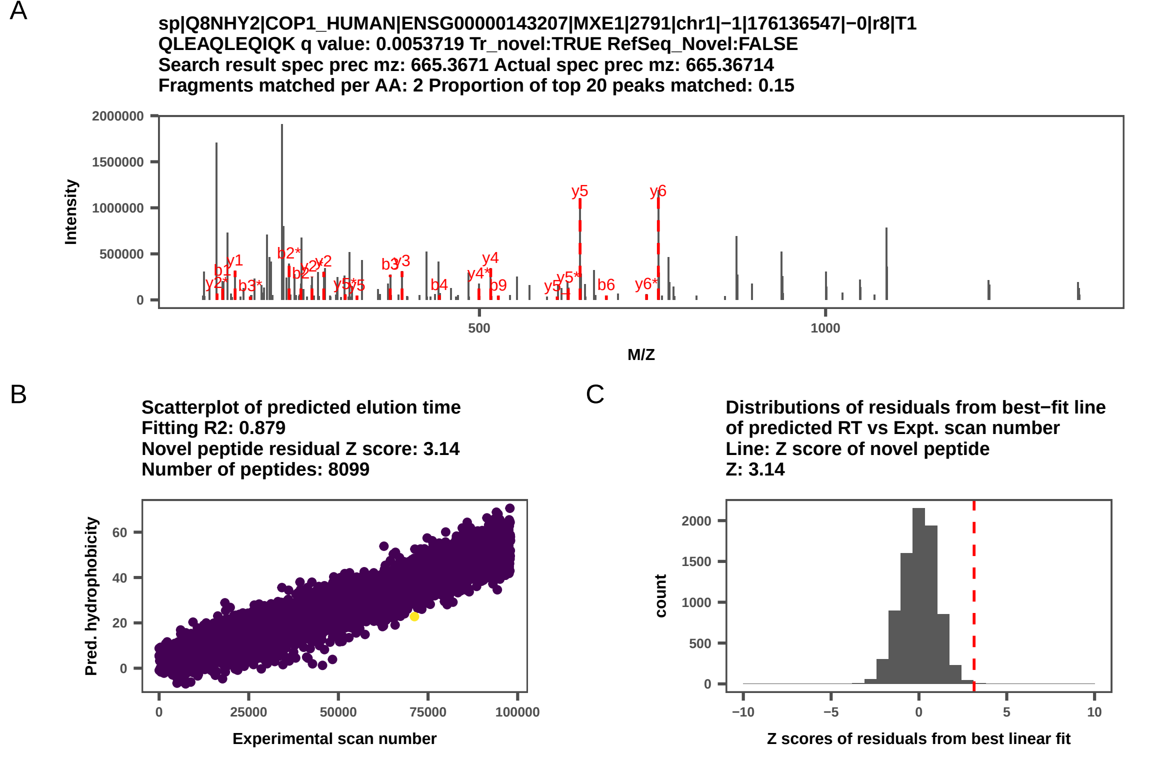
A
sp|Q8NHY2|COP1_HUMAN|ENSG00000143207|MXE1|2791|chr1|−1|176136547|−0|r8|T1
QLEAQLEQIQK q value: 0.0053719 Tr_novel:TRUE RefSeq_Novel:FALSE
Search result spec prec mz: 665.3671 Actual spec prec mz: 665.36714
Fragments matched per AA: 2 Proportion of top 20 peaks matched: 0.15
Intensity
2000000
1500000
1000000
500000
0
M/Z
500
1000
y5
y6
y1
b1
y2*
b3*
b2*
b2
y2*
y2
y5*
y5
b3
y3
b4
y4
y4*
b9
y5
y5*
b6
y6*
B
Scatterplot of predicted elution time
Fitting R2: 0.879
Novel peptide residual Z score: 3.14
Number of peptides: 8099
Pred. hydrophobicity
60
40
20
0
0
25000
50000
75000
100000
Experimental scan number
C
Distributions of residuals from best−fit line
of predicted RT vs Expt. scan number
Line: Z score of novel peptide
Z: 3.14
count
2000
1500
1000
500
0
−10
−5
0
5
10
Z scores of residuals from best linear fit
